# Changes in Trimethylamine-N-oxide Levels in Obese Patients following Laparoscopic Roux-en-Y Gastric Bypass or Sleeve Gastrectomy in a Korean Obesity Surgical Treatment Study (KOBESS)

**DOI:** 10.3390/jcm10215091

**Published:** 2021-10-29

**Authors:** Soo Jin Lee, Young Suk Park, Yong-Jin Kim, Sang-Uk Han, Geum-Sook Hwang, Yeyoung Han, Yoonseok Heo, Eunyoung Ha, Tae Kyung Ha

**Affiliations:** 1 Department of Nuclear Medicine, Hanyang University Medical Center, Seoul 04763, Korea; suji76@hanmail.net; 2 Department of Surgery, Seoul National University Bundang Hospital, Seongnam 13620, Korea; youngsukmd@gmail.com; 3 Department of Surgery, H Plus Yangji Hospital, Seoul 08779, Korea; yjgs1997@gmail.com; 4 Department of Surgery, Ajou University Hospital, Suwon 16499, Korea; hansu@ajou.ac.kr; 5 Integrated Metabolomics Research Group, Western Seoul Center, Korea Basic Science Institute, Seoul 24341, Korea; gshwang@kbsi.re.kr (G.-S.H.); tblove519@gmail.com (Y.H.); 6 Department of Chemistry, Sungkyunkwan University, 2066 Seobu-ro, Jangan-gu, Suwon-si 16419, Korea; 7 Department of Surgery, College of Medicine, Inha University, Incheon 22212, Korea; gshur@inha.ac.kr; 8 Department of Biochemistry, School of Medicine, Keimyung University, Daegu 42601, Korea; 9 Department of Surgery, Hanyang University College of Medicine, 222 Wangsimni-ro, Seongdong-gu, Seoul 04764, Korea

**Keywords:** trimethylamine N-oxide, cardiovascular disease, obesity, bariatric surgery, diabetes mellitus

## Abstract

Trimethylamine N-oxide (TMAO), a gut microbe-dependent metabolite, has been implicated as a novel risk factor for cardiovascular events related to obesity and type 2 diabetes mellitus (T2DM). The aim of the study was to test the hypothesis if TMAO is associated with the reduction of cardiovascular disease in the Korean obese patients who underwent bariatric surgery. From a subgroup of a multicenter, nonrandomized, controlled trial, titled KOBESS, 38 obese patients, 18 with and 20 without T2DM, who underwent Roux-en-Y gastric bypass (RYGB) or sleeve gastrectomy (SG) were investigated. Bariatric surgery is indicated for Korean patients with a body mass index (BMI) ≥ 35 kg/m^2^ or for Korean patients with a BMI ≥ 30 kg/m^2^ who have comorbidities. Serum levels of TMAO and its precursors, betaine, carnitine, and choline were measured before and six months after bariatric surgery. The levels of TMAO and its precursors did not differ between obese patients with T2DM and non-T2DM at baseline. However, TMAO increased more than twofold in patients with T2DM after RYGB surgery, but not in patients without T2DM. Choline levels were decreased by half in all patients after RYGB. In patients with T2DM who underwent SG, TMAO, betaine, and carnitine levels did not change after the surgery. Furthermore, in obese patients who underwent bariatric surgery, increased TMAO levels were associated with both T2DM and RYGB, while reduced choline levels were associated with RYGB. These associations need to be further elucidated in follow-up studies to gain further insights into the relationship between TMAO levels and bariatric surgery outcomes.

## 1. Introduction

Obesity is an ever-growing disease that is strongly associated with metabolic syndrome, characterized by insulin resistance, hyperglycemia, hyperlipidemia, and hypertension. Patients with obesity and diabetes mellitus (DM) are at an increased risk of cardiovascular morbidity and mortality. Several clinical trials have shown the significant effects of bariatric surgery, including weight loss, improvements in serum glucose control, and reduced risk of cardiovascular diseases [1,2].

Trimethylamine N-oxide (TMAO), a gut microbe-dependent metabolite, is a small organic compound derived from dietary choline, betaine, and L-carnitine through metabolic processes of gut microbiota and subsequently by hepatic flavin monooxygenases [3,4,5]. Evidence suggests that TMAO induces platelet hyperactivity and thrombosis, thereby increasing the atherosclerotic burden [6]. These findings are replicated in other clinical studies that showed an association between elevated TMAO levels and an increased risk of atherosclerosis and cardiovascular disease (CVD) [7,8]. Moreover, prospective cohort studies have shown that increased TMAO levels could predict an elevated risk of major adverse events, such as myocardial infarction, stroke, or death [8,9,10]. In addition, increased levels of TMAO are strongly associated with obesity and DM [11,12]. Recent observational studies have reported TMAO levels to be elevated after bariatric surgery [13,14].

As expected, bariatric surgery changes the composition of the gut microbiome owing to the characteristics of the surgical procedures involving the reconstruction of the small intestine [15]. However, TMAO levels were reported to be increased in Norwegians after bariatric surgery [13]. Given the well-established beneficial effects of bariatric surgery on attenuating the risks of CVDs, increased level of TMAO, a molecule that has been suggested as a risk factor for CVD, after bariatric surgery is conflicting and contradictory. To the best of our knowledge, as of today, the impact of bariatric surgery on TMAO change in Asians has not been reported.

Thus, in this prospective study, we investigated the levels of TMAO and its precursors to elucidate the association between TMAO and the risk of CVD after bariatric surgery. We subdivided patients in this study according to the presence or absence of DM and the types of bariatric surgery.

## 2. Material and Methods

### 2.1. Patients and Study Design

The current study is part of a clinical trial entitled Korean Obesity Surgical Treatment Study (KOBESS), registered at www.ClinicalTrials.gov, accessed on 10 September 2021 (NCT03100292) [16]. KOBESS is a prospective, multicenter, nonrandomized, controlled study of obese Korean patients who underwent primary sleeve gastrectomy (SG) or Roux-en-Y gastric bypass (RYGB). All patients were recruited between August 2016 and April 2019. Patients with a body mass index (BMI) ≥ 35 kg/m^2^ or a BMI 30.0–34.9 kg/m^2^ and obesity-related comorbidities, such as DM, hypertension, or hyperlipidemia, were considered eligible for KOBESS. The protocol was approved by the Institutional Review Board of each clinical center (approval number for the coordinating investigator: 000000 2016-06-015), and informed written consent was obtained from all participants. Patients who had complete results for laboratory tests conducted at baseline and six months after surgery were enrolled. Patients with serum samples of less than 50 μL, which was considered insufficient volume for analysis, were excluded from the study. Of 64 KOBESS patients, 38 obese patients, 18 with and 20 without type 2 DM (T2DM), were enrolled and investigated in the current study (Supplementary Figure S1). Seventeen patients (7 with T2DM and 10 without T2DM) underwent RYGB, and 21 patients (11 with T2DM and 10 without T2DM) underwent SG.

### 2.2. Anthropometric and Laboratory Assessments

Patients were assessed for anthropometry and blood chemistry at baseline two weeks before surgery. Follow-up examinations were performed six months after surgery. Anthropometric data and laboratory test results were recorded for each of the 38 patients six months after bariatric surgery. Height, weight, sex, systolic and diastolic blood pressure (mmHg), and BMI (kg/m^2^) were measured and recorded. Fasting blood samples were collected using a standard venipuncture and stored at –70 °C. Laboratory tests, including complete blood count, fasting plasma glucose, glycosylated hemoglobin (HbA1c), lipid profile (triglycerides, total cholesterol, high-density lipoprotein cholesterol [HDL-C], low-density lipoprotein cholesterol [LDL-C]), liver panel [aspartate aminotransferase (AST), alanine aminotransferase (ALT), γ-glutamyl transpeptidase (GTP), alkaline phosphatase (ALP)], renal panel [creatinine, blood urea nitrogen (BUN), uric acid (mg/dL)], ferritin, iron, vitamin B, and folate were performed. The diagnosis of T2DM at baseline was defined according to a previous diagnosis; HbA1c ≥ 6.5%, fasting serum glucose when fasting for more than 8 h ≥ 126 mg/dL, or serum glucose after 75 g oral glucose tolerance test ≥ 200 mg/dL.

### 2.3. Metabolomic Analysis

For targeted quantitative analysis, we performed ultra-high-performance liquid chromatography/triple quadrupole mass spectrometry (UPLC/TQ-MS) analysis. Prior to analysis, 20 μL of serum sample was extracted using 80 μL of methanol, and the aqueous supernatant was diluted with 20% acetonitrile (v/v) containing 5 ng/mL betaine-d11, an internal standard. UPLC/TQ-MS analysis was performed on an Agilent 1290 Infinity LC and an Agilent 6495 triple quadrupole MS system equipped with an Agilent Jet Stream electrospray ionization source (Agilent Technologies, USA). Chromatographic separation was carried out on an Acquity UPLC BEH amide column (2.1 mm × 50 mm, 1.7 μm; Waters) with a binary gradient system comprising 10 mM ammonium formate in water (solvent A) and acetonitrile (solvent B). The linear gradient elution was as follows: 0–1.0 min, 85% B; 1.0–2.5 min, 85–40% B; 2.5–3.0 min, 40% B; 3.0–3.1 min, 40–85% B; 3.1–5.1 min, 85% B. Quantification was performed in the multiple reaction monitoring mode using MS operation in positive ionization mode. Mass Hunter Workstation (Ver B.06.00, Agilent Technologies, USA) software was used for data acquisition and analysis. Metabolite analysis was performed on serum samples collected at baseline and six months after bariatric surgery from 37 patients. Data from one patient were excluded because of measurement failure.

### 2.4. Statistical Analysis

All data are expressed as the mean ± standard deviation (SD). A paired *t*-test or independent *t*-test was used to assess the difference in each variable between baseline and six months after surgery. A significance level of 0.05 was used. Statistical analyses were performed using commercial software packages (SPSS version 19; IBM, Chicago, IL, USA), and *p*-values less than 0.05 were considered to be statistically significant.

## 3. Results

### 3.1. Clinical Characteristics

As a subgroup of a prospective multicenter clinical trial, a total of 38 obese patients who underwent both bariatric surgery and laboratory tests conducted at baseline and six months after surgery were enrolled (Supplementary Figure S1). The baseline characteristics of the two groups divided by the prevalence of T2DM (18 patients with T2DM and 20 without T2DM) are presented in Table 1. At baseline, the mean age in the T2DM group was 8 years older (*p* = 0.034) and the HbA1c level was 2.2% higher than in the non-T2DM group (*p* < 0.001). In the T2DM group, BMI was slightly higher than that of non-T2DM (37.8 ± 5.9 kg/m^2^ vs. 40.1 ± 6.4 kg/m^2^), but there was no significant difference. There were no differences in sex, type of surgery, systolic blood pressure (SBP), diastolic blood pressure (DBP), total cholesterol, and triglyceride levels between the two groups. In the T2DM group, 7 (38.9%) patients underwent RYGB, and 11 (61.1%) underwent SG. In the non-T2DM group, 10 (50.0%) patients underwent RYGB, and 10 (50.0%) underwent SG. All surgical procedures were performed without any significant postoperative complications, and all patients received recommendations regarding dietary habits and lifestyle modifications and micronutrient supplementation (vitamin D, vitamin B_12_, multivitamin, calcium) as required.

Table 2 shows BMI and changes in serum biochemical indices six months after surgery for two groups. After bariatric surgery, BMI decreased significantly by more than 10 kg/m^2^ in both groups (both *p* < 0.001). In addition, both SBP and DBP decreased in the two groups, but the decrease was not significant. In patients with T2DM, HbA1c decreased significantly from 7.8% to 6.2% and mean value of serum glucose markedly decreased from 144 mg/dL to 105 mg/dL. Even in non-T2DM patients, mean value of serum glucose decreased from 102 mg/dL to 94 mg/dL (*p* = 0.003). AST, ALT and HDL-C were significantly improved in both groups (*p* < 0.05 for all). In addition, in non-T2DM patients, GTP, total cholesterol and triglyceride decreased significantly. Levels of BUN and uric acid of both groups remained unchanged. The level of ferritin decreased in both groups, and VitB_12_ decreased in T2DM after bariatric surgery.

### 3.2. Changes in Metabolites

**Figure 1:**
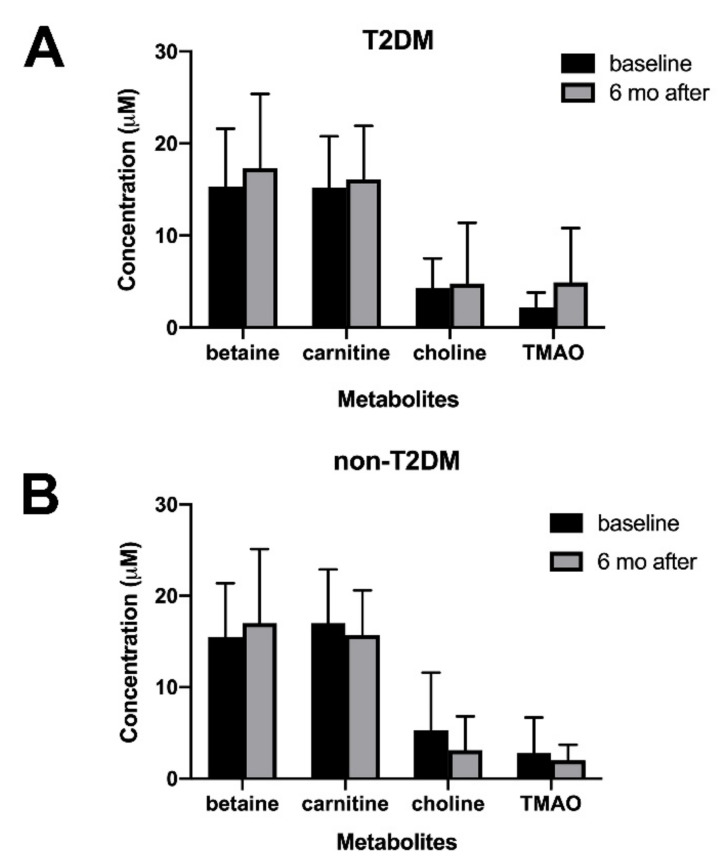
Serum levels of metabolites at baseline (black bars) and six months after bariatric surgery (gray bars) in (**A**) Type 2 diabetes mellitus (T2DM) and (**B**) non-T2DM patients. Abbreviation: T2DM, type 2 diabetes mellitus; TMAO. Trimethylamine-N-oxide.

Table 3 shows the serum levels of betaine, carnitine, choline, and TMAO at baseline and six months after bariatric surgery (RYGB and SG). Betaine, carnitine, and choline are the precursors of TMAO. Serum levels of TMAO and its precursors did not change at six months after surgery. (*p* > 0.05). When stratified by the presence and absence of T2DM, serum levels of TMAO, although not statistically significant (2.2 ± 1.6 vs. 4.9 ± 5.9 μM, *p* = 0.072), appeared increased in patients with T2DM (Figure 1 and Supplementary Table S1). The levels of betaine, carnitine, and choline remained unchanged after surgery in both the T2DM and non-T2DM groups.

We stratified the patients according to the type of surgery, RYGB and SG (Table 4). We found no difference in baseline levels of TMAO and its precursors. After surgery, TMAO, betaine, and carnitine levels did not significantly change in both the T2DM and non-T2DM groups. Intriguingly, we observed significantly decreased levels of choline (3.2 ± 2.1 µM at baseline, 1.7 ± 1.4 µM after six months, *p* = 0.004) after RYBG, but not after SG (*p* = 0.850).

Based on the increased level of TMAO in patients with T2DM, we stratified patients with T2DM and non-T2DM according to the type of bariatric surgery: T2DM with RYGB (n = 7), T2DM with SG (n = 11), non-T2DM with RYGB (n = 10), and non-T2DM with SG (n = 9). We also analyzed the levels of the metabolites (Table 5). Betaine and carnitine levels were not affected by the presence of T2DM or the type of surgery. Contrary to the levels of betaine and carnitine, those of choline and TMAO appeared to be influenced by the presence of T2DM or the type of surgery. In T2DM patients who underwent RYGB, the level of TMAO increased more than twofold (2.3 ± 1.5 to 5.5 ± 3.1 µM, *p* = 0.043). In contrast, in patients with non-T2DM, TMAO levels did not change in either the RYGB or SG groups. These results suggest a possible association of TMAO with T2DM and RYGB. We also observed that the level of choline was associated with RYGB. The level of choline was attenuated in non-T2DM patients who underwent RYGB (3.2 ± 2.0 to 2.1 ± 1.5 µM, *p* = 0.036). The mean level of choline, although not significant, decreased from 3.3 ± 2.5 to 1.2 ± 1.2 µM (*p* = 0.091) in T2DM patients who underwent RYGB.

## 4. Discussion

In our study of obese patients who underwent bariatric surgery, we observed that the serum levels of TMAO increased substantially in T2DM patients who underwent RYGB, while other precursor metabolites, betaine and carnitine, were not altered. We also observed that the level of choline decreased significantly in all patients who underwent RYGB. Meanwhile, there was no significant change in metabolites including TAMO in patients with SG regardless of diabetes.

In the current study, we observed that TMAO levels were particularly associated with T2DM and RYGB. Previous studies have reported that TMAO levels after bariatric surgery, particularly RYGB, are increased [13,14], but not after vertical banded gastroplasty [17]. Trøseid et al. reported that plasma levels of TMAO more than doubled compared to the preoperative level in 27 obese patients one year after RYGB (4.4 μM vs. 10.5 μM, *p* < 0.001) [13]. Tremaroli et al. observed increased levels of TMAO only in patients who underwent RYGB but not in those that underwent vertical-banded gastroplasty, nine years after bariatric surgery [18]. In addition, they did not observe differences in other metabolites, carnitine and betaine, between the control and surgery groups.

The mechanism underlying the increase in TMAO levels after RYGB surgery remains to be elucidated. One possibility is that this may be due to adaptive shifts in the gut microbiota. Studies have indicated that bariatric surgery produces a specific shift in the gut microbiota that persists for up to a decade after surgery and is different from the shifts related to dietary intervention for weight loss [15,18,19]. Li et al. observed a major shift in the gut phyla towards higher concentrations of Proteobacteria (52-fold), lower concentrations of Firmicutes (4.5-fold), and Bacteroidetes (twofold) in a non-obese RYGB rat model compared with sham-operated rats [20]. Tremaroli et al. suggested that the increased level of TMAO after bypass surgery might be the consequence of less anaerobic metabolism in the intestine after bypass surgery, a hypothesis that is supported by the broad increase in facultative anaerobes in the intestine after RYGB [18]. Supporting this hypothesis, a recent study indicated that Proteobacteria is the most important bacteria as it encodes the *cutC* gene that codes for choline to trimethylamine (TMA)-mediating enzyme, choline TMA-lyase [21]. Thus, adaptive shifts in the gut microbiota of RYGB surgery may be responsible for the increased level of TMAO. In the current study, however, contrary to the more than doubled level in T2DM patients who underwent RYGB, we did not observe any change in TMAO levels in non-T2DM patients who underwent RYGB. This result clearly does not support the hypothesis that adaptive shifts in the gut microbiota are responsible for the increase in TMAO levels after RYGB surgery.

Another possible explanation for the increased TMAO level after RYGB surgery is that flavin-containing monooxygenase 3 (FMO3), the hepatic enzyme that produces TMAO, might be responsible for the increase in TMAO levels. Higher levels of TMAO are associated with T2DM [11,22]. A recent meta-analysis demonstrated a positive dose-dependent association between TMAO levels and an increased risk for T2DM [23]. The study indicated that the odds ratio for DM prevalence increased by 54% per 5 µM increment in serum TMAO levels. An interesting recent study showed that FMO3 is suppressed by insulin and increases in obese/insulin-resistant humans [24]. More intriguingly, a study reported that RYGB corrects fasting hyperinsulinemia in patients with T2DM [25]. Based on the above-referenced studies, decreased production of insulin during fasting after RYGB could contribute to the increased activity of FMO3, leading to increased levels of TMAO.

In addition to the increased level of TMAO in T2DM patients who underwent RYGB, we also observed that reduced choline levels were associated with RYGB. The decreased choline level is likely due to the rearrangement of the anatomy of the intestine rather than the presence of T2DM, since choline levels were significantly reduced in all patients who underwent RYGB. In addition, we did not observe any change in choline levels in patients who underwent SG, in which the anatomy of the intestine remains intact. Adaptive shifts in gut microbiota towards higher concentrations of bacteria, such as Proteobacteria, that actively convert choline into TMA could explain the decreased choline level after RYGB.

This study is limited to the insufficient power of statistical significance. However, insufficient power of statistical significance does not exclude the possible effect of TMAO on cardiovascular disease. The aim of the study was to test the hypothesis if TMAO is associated with the reduction of cardiovascular disease in the Korean obese patients who underwent bariatric surgery. The prevalence of obesity in Korea steadily increased and the incidence of metabolic disease also concomitantly increased. Bariatric surgery has been reimbursed by National Health Insurance Service in Korea since 2019. The indication of bariatric surgery in Korea is different from that in Western countries. Bariatric surgery is indicated for Korean patients with BMI ≥ 35kg/m^2^ or for Korean patients with BMI ≥ 30kg/m^2^ who have comorbidities (T2DM, hypertension, dyslipidemia, obstructive sleep apnea etc.). Therefore, these data cannot be applied to the patients in Western countries. Asian people have similar characteristics and comorbidity. Based on this preliminary study, a large clinical study is underway to determine if TMAO can be a risk factor for cardiovascular disease (Clinical trials No. NCT04554758).

To the best of our knowledge, this is the first prospective study to reveal that the increase in TMAO levels after RYGB is associated with T2DM. The current study is a relatively short-term study of six months. Given the conflicting facts that high TMAO levels are implicated in CVD, while RYGB surgery reduces the risk of CVD, further prospective long-term studies are necessary to gain further insights into the relationship between TMAO levels and bariatric surgery outcomes.

## Figures and Tables

**Table 1:** Baseline characteristics of patients with type 2 diabetes mellitus (T2DM) and non-T2DM.

	T2DM (n = 18)	Non-T2DM (n = 20)	*p* Value
Gender (male: female)	13:5	14:6	0.884
Age (years)	43.8 ± 13	35.4 ± 11	0.034
BMI (kg/m^2^)	37.8 ± 5.9	40.1 ± 6.4	0.271
RYGB:SG	7:11	10:10	0.505
HbA1c (%)	7.8 ± 1.6	5.6 ± 0.3	<0.001
SBP (mmHg)	131 ± 13	140 ± 20	0.123
DBP (mmHg)	80 ± 12	85 ± 18	0.324
Total cholesterol (mg/dL)	192 ± 41	183 ± 28	0.420
Triglyceride (mg/dL)	213 ± 213	152 ± 69	0.234

Data are presented as the mean ± standard deviation (SD) or number (n). T2DM, type 2 diabetes mellitus; BMI, body mass index; RYGB, Roux-en-Y gastric bypass; SG, sleeve gastrectomy, HbA1c, glycosylated hemoglobin; SBP, systolic blood pressure; DBP, diastolic blood pressure.

**Table 2:** Serum indices at baseline and six months after bariatric surgery in patients with T2DM and non-T2DM.

	T2DM (n = 18)			non-T2DM (n = 20)		
	Baseline	Six Months after Surgery	*p* Value	Baseline	Six Months after Surgery	*p* Value
Age (years)	43.8 ± 12.9			35.4 ± 10.8		
BMI (kg/m^2^)	37.8 ± 5.9	27.5 ± 5.2	<0.001	40.1 ± 6.4	29.9 ± 5.2	<0.001
SBP (mmHg)	131 ± 13	124 ± 20	0.206	140 ± 20	133 ± 17	0.250
DBP (mmHg)	80 ± 12	78 ± 13	0.626	85 ± 18	80 ± 17	0.443
HbA1c (%)	7.8 ± 1.6	6.2 ± 1.1	<0.001	5.6 ± 0.3	-	-
Glucose (mg/dL)	144 ± 50	105 ± 21	0.009	102 ± 11	94 ± 6	0.003
AST (IU/L)	39.3 ± 22	22.8 ± 6.1	0.004	34.8 ± 18	20.3 ± 8.5	0.002
ALT (IU/L)	52.3 ± 37	22.5 ± 12	0.005	57.7 ± 37	19.4 ± 11.8	<0.001
GTP (IU/L)	47.1 ± 27	27.9 ± 29.8	0.086	38.4 ± 21	15.1 ± 8.7	<0.001
ALP (IU/L)	85.3 ± 22	82.1 ± 26.5	0.726	72.2 ± 17	71.4 ± 13	0.713
Total cholesterol (mg/dL)	192 ± 41	180 ± 36	0.060	183 ± 28	166 ± 26	0.012
Triglyceride (mg/dL)	213 ± 213	122 ± 54	0.079	152 ± 69	94 ± 32	<0.001
HDL-C (mg/dL)	47.1 ± 8.3	51.8 ± 10	0.030	47.5 ± 13	54.2 ± 14	0.001
LDL-C (mg/dL)	116 ± 27	109 ± 28	0.292	116 ± 30	103 ± 21	0.054
Creatinine (mg/dL)	0.79 ± 0.22	0.72 ± 0.20	0.693	0.75 ± 0.19	0.72 ± 0.15	0.287
BUN (mg/dL)	13.7 ± 7.2	13.8 ± 6.5	0.877	12.2 ± 4.0	11.3 ± 3.3	0.411
Uric acid (mg/dL)	5.5 ± 1.5	5.1 ± 1.1	0.127	6.0 ± 1.7	5.0 ± 1.3	0.004
Ferritin	165.8 ± 158.7	107.2 ± 117.8	0.011	157.8 ± 146	101.2 ± 102	0.010
Iron	101 ± 27	100 ±30	0.863	87 ± 39	105 ± 36	0.050
VitB12	621 ± 305	516 ± 218	0.040	475 ± 204	433 ± 209	0.180
Folate	9.06 ± 4.6	8.94 ± 5.3	0.921	6.61 ± 3.7	6.75 ± 4.9	0.861

Data are presented as the mean ± standard deviation. T2DM, type 2 diabetes mellitus; BMI, body mass index; SBP, systolic blood pressure; DBP, diastolic blood pressure; HbA1c, glycosylated hemoglobin; AST, aspartate aminotransferase; ALT, alanine aminotransferase; GTP, γ-glutamyl transpeptidase; ALP, alkaline phosphatase; HDL-C, high-density lipoprotein cholesterol; LDL-C, low-density lipoprotein cholesterol.

**Table 3:** Serum metabolites at baseline and six months after bariatric surgery.

	Baseline	Six Months after	Δ	*p* Value
Betaine (µM)	15.4 ± 6.1	17.2 ± 7.9	1.8 ± 6.7	0.116
Carnitine (µM)	16.0 ± 5.7	15.9 ± 5.3	−0.095 ± 4.3	0.894
Choline (µM)	4.8 ± 5.0	3.9 ± 5.3	−9.0 ± 6.4	0.396
TMAO (µM)	2.5 ± 3.0	3.5 ± 4.5	0.95 ± 5.4	0.292

Data are presented as the mean ± standard deviation. Δ, difference between baseline and six months after surgery; TMAO, trimethylamine-N-oxide.

**Table 4:** Serum indices at baseline and after six months in subjects who underwent Roux-en-Y gastric bypass (RYGB) and sleeve gastrectomy (SG).

	RYGB (n = 17)			SG (n = 21) *			
	Baseline	Six Months after	*p* value	Baseline	Six Months after	*p* Value	*p*^§^ Value
Age (years)	41.2 ± 13.4			38.0 ± 11.7			0.444
Male: Female	6:11			5:16			0.451
T2DM: non-T2DM	7:10			11:10			0.505
BMI (kg/m^2^)	39.2 ± 5.9	28.1 ± 3.8	<0.001	38.9 ± 6.5	28.4 ± 6.2	<0.001	0.865
SBP (mmHg)	128 ± 10	133 ± 23	0.450	141 ± 20	126 ± 14	0.002	0.021
DBP (mmHg)	78 ± 8	83 ± 20	0.319	86 ± 18	76 ± 8	0.060	0.125
HbA1c (%)	8.5 ± 1.1 (n = 7)	6.9 ± 1.3	0.002	7.5 ± 1.8 (n = 10)	5.8 ± 0.7	0.007	0.394
Glucose (mg/dL)	126 ± 29	105 ± 16	0.007	119 ± 48	95 ± 15	0.028	0.621
Betaine (µM)	14.5 ± 6.9	17.1 ± 8.6	0.092	16.2 ± 5.4	17.2 ± 7.6	0.529	0.411
Carnitine (µM)	14.8 ± 6.7	13.9 ± 5.9	0.405	17.0 ± 4.7	17.5 ± 4.1	0.588	0.268
Choline (µM)	3.2 ± 2.1	1.7 ± 1.4	0.004	6.2 ± 6.3	5.8 ± 6.5	0.850	0.059
TMAO (µM)	2.9 ± 3.8	3.5 ± 2.7	0.613	2.2 ± 2.0	3.5 ± 5.6	0.365	0.492

* Metabolite data from 20 patients. ^§^ Comparison of baseline values between the two groups. Data are presented as the mean ± standard deviation or number (n). RYGB, Roux-en-Y gastric bypass; SG, sleeve gastrectomy; T2DM, type 2 diabetes mellitus; BMI, body mass index; SBP, systolic blood pressure; DBP, diastolic blood pressure; HbA1c, glycosylated hemoglobin; TMAO, trimethylamine-N-oxide.

**Table 5:** Changes in metabolites according to diabetes and surgery type.

	T2DM with RYGB (n = 7)			T2DM with SG (n = 11)			Non-T2DM with RYGB (n = 10)			Non-T2DM with SG (n = 9)		
	Baseline	Six Months after	*p* Value	Baseline	Six Months after	*p* Value	Baseline	Six Months after	*p* Value	Baseline	Six Months after	*p* Value
Betaine (µM)	13.3 ± 7.5	15.1 ± 2.9	0.398	16.5 ± 5.5	18.8 ± 10.0	0.477	15.4 ± 6.8	18.5 ± 11.0	0.241	15.7 ± 5.5	15.3 ± 2.1	0.553
Carnitine (µM)	12.7 ± 7.1	13.1 ± 6.6	0.866	16.8 ± 4.0	18.1 ± 4.4	0.328	16.3 ± 6.3	14.6 ± 5.7	0.260	17.2 ± 5.7	16.9 ± 3.9	0.953
Choline (µM)	3.3 ± 2.5	1.2 ± 1.2	0.091	5.0 ± 3.5	7.1 ± 7.6	0.656	3.2 ± 2.0	2.1 ± 1.5	0.036	7.6 ± 8.6	4.3 ± 5.0	0.441
TMAO (µM)	2.3 ± 1.5	5.5 ± 3.1	0.043	2.2 ± 1.7	4.6 ± 7.3	0.306	3.3 ± 4.9	2.0 ± 1.1	0.878	2.2 ± 2.4	2.1 ± 2.2	0.813

Data are presented as the mean ± standard deviation or number (n). RYGB, Roux-en-Y gastric bypass; SG, sleeve gastrectomy; T2DM, type 2 diabetes mellitus; TMAO, trimethylamine-N-oxide.

## Data Availability

The data described in the manuscript, code book, and analytic code will be made available upon request pending application and approval.

## Supplementary Materials

The following are available online at https://www.mdpi.com/article/10.3390/jcm10215091/s1, Figure S1: Flow chart of the KOBESS study. Table S1: The changes in metabolites at baseline and six months in subjects with T2DM and non-T2DM.
