# Supplementary material for: Changes in Trimethylamine-N-oxide Levels in Obese Patients following Laparoscopic Roux-en-Y Gastric Bypass or Sleeve Gastrectomy in a Korean Obesity Surgical Treatment Study (KOBESS)
Source: J Clin Med. 2021 Oct 29;10(21):5091. doi: 10.3390/jcm10215091 (PMC8584728; doi:10.3390/jcm10215091)
Supplement: Supplementary file 1 [file jcm-10-05091-s001.zip › jcm-1398786-supplementary.pdf]

**Table S1.** The changes in metabolites at baseline and 6 months in subjects with T2DM and non-T2DM.

| | **T2DM (n=18)** | | | **Non-T2DM (n=20)** | | | |
|---|---|---|---|---|---|---|---|
| | **Baseline** | **Six months after** | ***P* value** | **Baseline** | **Six months after** | ***P* value** | ***P** value** |
| Betaine (μM) | 15.3 ± 6.3 | 17.3 ± 8.1 | 0.235 | 15.5 ± 6.0 | 17.0 ± 8.1 | 0.325 | 0.897 |
| Carnitine (μM) | 15.2 ± 5.6 | 16.1 ± 5.8 | 0.410 | 16.7 ± 5.9 | 15.7 ± 4.9 | 0.247 | 0.429 |
| Choline (μM) | 4.3 ± 3.2 | 4.8 ± 6.6 | 0.732 | 5.3 ± 6.3 | 3.1 ± 3.7 | 0.192 | 0.574 |
| TMAO (μM) | 2.2 ± 1.6 | 4.9 ± 5.9 | 0.072 | 2.8 ± 3.9 | 2.0 ± 1.7 | 0.470 | 0.581 |

*Comparison of baseline values between two groups, Data presented as mean ± standard deviation.
T2DM type 2 diabetes mellitus; BMI body mass index; TMAO trimethylamine-N-oxide

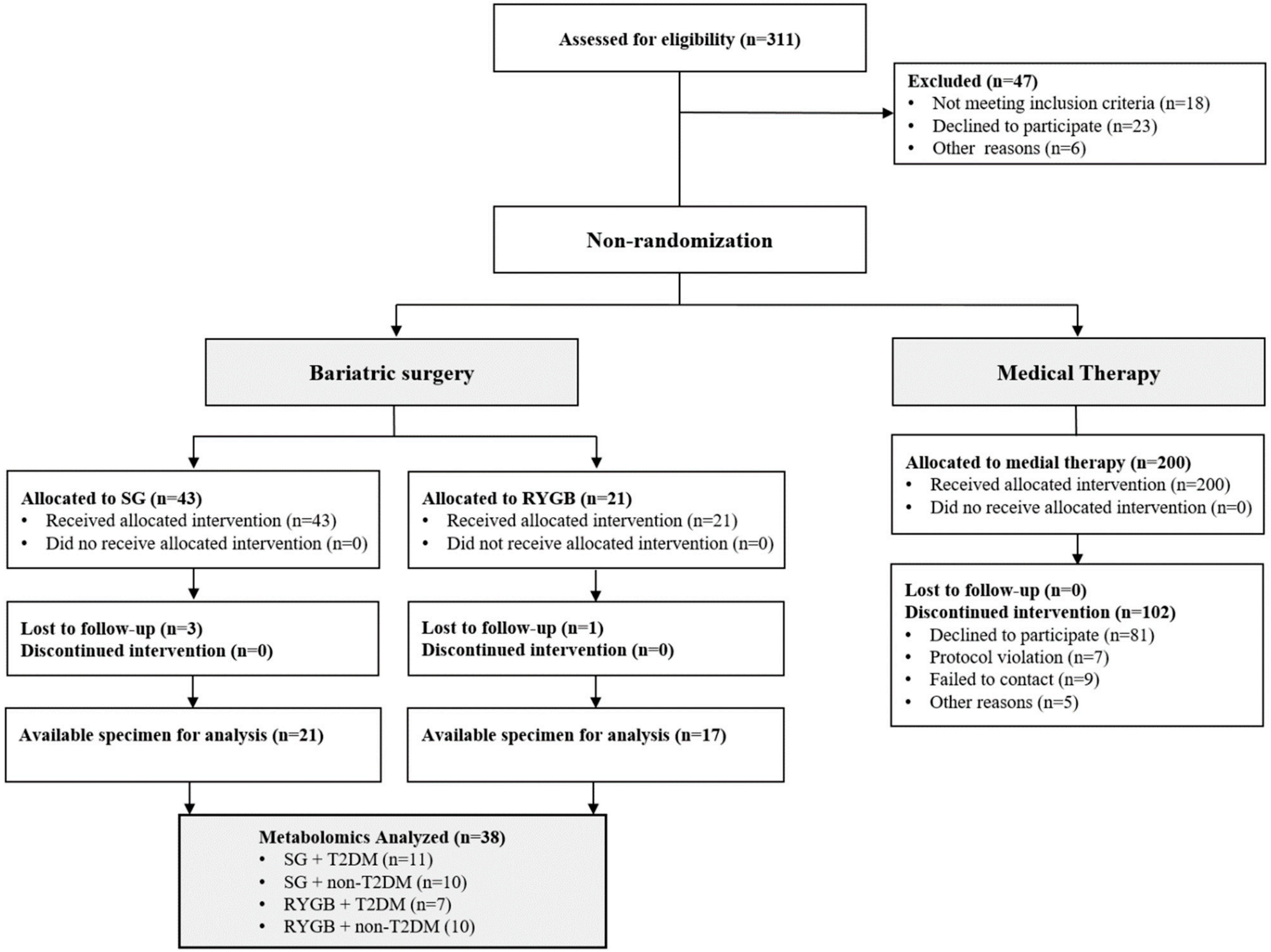


**Fig. S1** Flow chart of the KOBESS study
